# Mechanical Properties of Porous Structures for Dental Implants: Experimental Study and Computational Homogenization

**DOI:** 10.3390/ma14164592

**Published:** 2021-08-16

**Authors:** Aleš Jíra, Michal Šejnoha, Tomáš Krejčí, Jan Vorel, Luboš Řehounek, Guido Marseglia

**Affiliations:** 1 Department of Mechanics, Faculty of Civil Engineering, Czech Technical University in Prague, 166 29 Prague, Czech Republic; jira@fsv.cvut.cz (A.J.); sejnom@fsv.cvut.cz (M.Š.); krejci@fsv.cvut.cz (T.K.); jan.vorel@fsv.cvut.cz (J.V.); lubos.rehounek@fsv.cvut.cz (L.Ř.); 2 High Technical School of Architecture, University of Seville, 41012 Sevilla, Spain; 3 Instituto de Matemáticas de la Universidad de Sevilla, University of Seville, 41012 Sevilla, Spain

**Keywords:** porous material, titanium trabecular and gyroid structures, selective laser melting, mechanical properties, homogenization, FEM, X-FEM, dental implant

## Abstract

A combined experimental and numerical study on titanium porous microstructures intended to interface the bone tissue and the solid homogeneous part of a modern dental implant is presented. A specific class of trabecular geometries is compared to a gyroid structure. Limitations associated with the application of the adopted selective laser melting technology to small microstructures with a pore size of 500 μm are first examined experimentally. The measured effective elastic properties of trabecular structures made of Ti6Al4V material support the computational framework based on homogenization with the difference between the measured and predicted Young’s moduli of the Dode Thick structure being less than 5%. In this regard, the extended finite element method is promoted, particularly in light of the complex sheet gyroid studied next. While for plastic material-based structures a close match between experiments and simulations was observed, an order of magnitude difference was encountered for titanium specimens. This calls for further study and we expect to reconcile this inconsistency with the help of computational microtomography.

## 1. Introduction

Reducing high interface stresses (possibly causing interface debonding and eventual implant loosening) and minimizing the effect of stress shielding (a generally accepted factor of bone mass reduction) are two factors that drive the research efforts in the development of novel, more reliable implants [1,2,3]. While promoting more flexible implants to reduce stress shielding and consequently long-term bone loss seems reasonable, their application may generate inadmissible interface stresses at some locations of the bone–implant interface [2,4]. It is therefore the implant stiffness to bone stiffness ratio which deserves particular attention [5]. In this regard, an application of porous microstructures on the outer part of a stiff implant as a stabilizing element for relatively compliant human bone has attracted considerable interest, particularly when potentially designing implants tailored to patient-specific conditions [6] thus complying with the current trend in bioengineering [7,8].

A rapid boom in the design and modeling of porous microstructures, focusing on bone implants [9,10,11], has been observed with a recently developed additive manufacturing (AM) technique allowing for the production of porous titanium microstructures via 3D printing [12]. Either selective electron beam melting (SEBM) [10,13,14] or selective laser melting (SLM) [15,16] is typically adopted to fabricate microstructures of variable complexity, including both trabecular [10,17,18] and gyroid [9,11,14] types of cellular structures. The latter technique has been selected in this study owing to a fruitful collaboration with ProSpon, Ltd. which has successfully exploited SLM in a variety of biomedical areas, including production of implants and medical devices in orthopedics, traumatology, and surgery applications.

An overarching objective is to allow for an adequate osseointegration and tissue regeneration not only on the implant surface but also inside the porous scaffold, thus improving the bone–implant bonding properties and overall implant stability [19]. However, identifying an optimum pore size to facilitate the ingrowth of bone cells [20] while maintaining the desired mechanical properties remains a challenge. Considering the tissue growth only, it was observed in [21] that the maximum bond strength between the implant porous structure and the bone corresponds to a pore size in the range of 50–400 μm [12,22]. A series of in vitro tests on titanium implants with drilled cylindrical channels performed in [23] suggested a pore size of 600 μm to yield the highest rate of bone cell ingrowth. It was noted in [12] that this quantity evolves over time, promoting small pores up to 500 μm at an early stage of bone tissue ingrowth, while there is a higher bone cell density in bigger pores of 800–1000 μm for longer intervals. This issue deserves particular attention in applications of small dental implants where limitations of 3D printing to produce stable microstructures free of solid phase discontinuities [18] play a crucial role.

An extensive experimental campaign appears to be a natural way of assessing the mechanical properties of intended porous microstructures both within and beyond the elastic limits. A well-designed parametric study can be carried out to identify sensitivity of the selected microstructural details, including the shape and size of pores, various geometrical details of the solid phase, and the load and boundary conditions in the overall response both at the level of a cellular structure and an implant [24]. Although eventually irreplaceable, purely experimental research may prove expensive when searching for optimal or even patient-specific designs. To this goal, a computational approach is a suitable alternative [25,26]. In the framework of trabecular or gyroid structures, the interested reader is referred to [9,10,11,17,27], to cite a few. In this regard, triply periodic minimal surface (TPMS) structures have often been examined owing to their extraordinary mechanical properties that are easily tailored, through simple geometrical adjustments, to those of human trabecular bone [28]. As they possess periodicity in three mutually perpendicular directions [29] they represent a suitable candidate for computational homogenization [30,31,32,33,34] and multiscale modeling [35,36,37].

Both aspects of this research will be addressed herein, aiming at potential applications of porous microstructures to dental implants [1,38]. We intend to:

- Propose, design, and experimentally examine titanium porous specimens manufactured via the SLM 3D printing technique to acquire the basic mechanical properties in tension and compression of both trabecular and gyroid microstructures. We turn our attention to the production of specimens with a minimum number of internal flaws of both types of microstructures. This step exposes potential limitations of 3D printing of small dental implants.
- Examine the standard homogenization approach presented in the framework of 1st order computational homogenization to replace an expensive full-scale analysis of actual specimens. Limiting our attention to elasticity, several periodic unit cells corresponding to experimentally tested specimens are analyzed to identify the actual material symmetry of a given microstructure. Proving the applicability of this approach by comparing experimental measurements and numerical predictions represents an important step towards advanced nonlinear multiscale analyses of these complex microstructures.

## 2. Materials and Methods

Several trabecular and gyroid morphologies were examined, both experimentally (Section 2.1 and Section 3.1) and computationally (Section 2.2 and Section 3.2). The production feasibility with emphasis on dental implants was examined first. The resulting microstructures were mechanically tested to acquire basic data exploited in the validation step of the adopted computational homogenization. This latter technique is expected to provide an efficient tool when searching for optimal, patient-specific microstructures without performing time-consuming and expensive laboratory measurements, at least at the initial stage of design of a particular implant.

### 2.1. Experimental Program

Both trabecular and gyroid specimens were produced using the SLM technique (see also [6] for similar applications). The raw material adopted was a Ti6Al4V powder (Rematitan CL) with a maximum grain size of 63 μm supplied by the Dentaurum medical technology manufacturer. All specimens were manufactured in cooperation with ProSpon, Ltd. (Kladno, Czech Republic) employing the M2 Cusing machine. The 3D printing was carried out in argon atmosphere with 0.5% oxygen maintained in a welding chamber. The printed specimens were then heat treated (gradual heating up to 840 °C in 4 h—maintained at 840 °C for 2 h—cooled down to room temperature) in vacuum to relieve internal tension.

**Figure 1:**
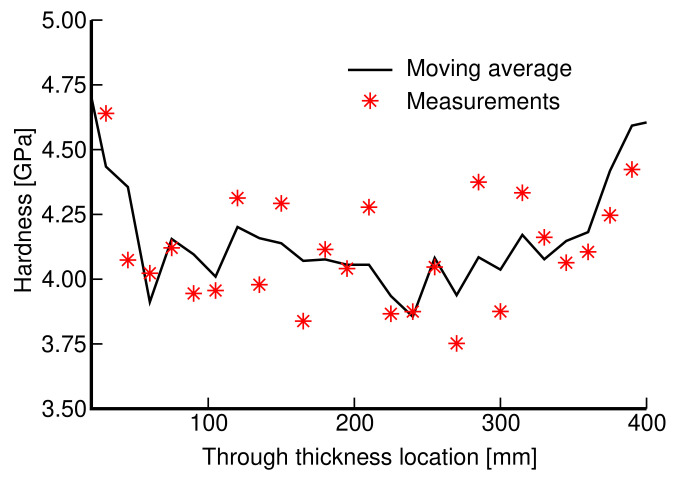
Variation in microhardness throughout the wall thickness.

Prior to mechanical testing, the specimens’ load-bearing elements (struts and walls in trabecular and gyroid microstructures, respectively) were subjected to nanoindentation to obtain the Young’s modulus of the bulk material. The tested specimen was embedded into an epoxy resin and upon curing the indented surface was ground and polished to obtain as smooth a surface as possible. A sufficiently dense indentation map was used to explore an expected variability in stiffness depending on the location and loading directions potentially attributed to a layerwise process of 3D printing. However, the resulting variation in the reduced modulus [39,40] confirmed an essentially isotropic response independent of the location with the values of $E_{r}$ in the range of 114–128 GPa which is considered sufficiently close to the value of 115 GPa provided by the manufacturer and comparable to the values observed for conventionally made Ti6Al4V-based implants [41]. Additionally, the measured microhardness, found in the range of 3.9–4.7 GPa, matches quite well with the values reported in the literature (see, e.g., [6] and the references therein) and does not vary much throughout the wall thickness, as seen in Figure 1. These observations suggest that the SLM-based 3D printing does not alter the expected mechanical properties of the bulk material, thus promoting advancement towards mechanical characterization of actual porous microstructures.

#### 2.1.1. Fabrication of Trabecular and Gyroid Specimens

**Figure 2:**
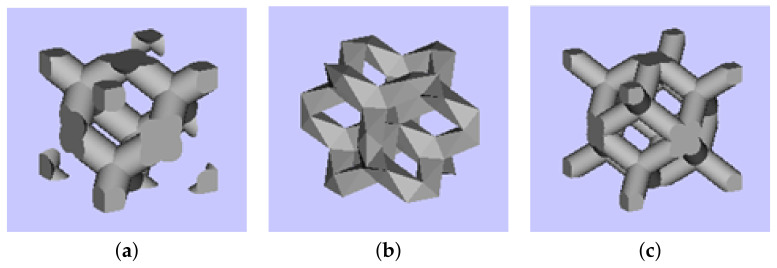
Basic cells of trabecular structures: (**a**) diamond (D30), relative density 30%, (**b**) Dode Thick (DT), (**c**) rhombic dodecahedron (RD30, relative density 30%).

The reliability of experimental results depends considerably on the quality of specimens. This issue is partially addressed here, starting with seemingly less complex morphologies of trabecular structures. Favoring production simplicity, we limit our attention to three basic cells (building blocks) plotted in Figure 2.

As the rate of osseointegration and consequently the required implant stability depend on the size of pores (d), we produced six types of specimens with the pore sizes ranging from 350 to 800 μm. Limitations of the available printing device did not allow for manufacturing specimens with a strut thickness $\left(\delta^{s}\right)$ less than 200 μm. Although not the primary objective, we kept the overall porosity ($n^{m},n^{V}$), given the desired pore size, of the specimens comparable by conveniently adjusting the strut thickness thus arriving at basic units of a variable size (L). The basic data are listed in Table 1. Therein, we introduce two types of porosities representing a theoretical porosity $n^{V}$ derived from the original CAD models and the actual one denoted as $n^{m}$ and derived for a printed specimen from

(1) $$n^{m}=1-\frac{m-V\rho_{m}}{Ah\rho_{m}},$$

where m,V,A,h are the specimen mass, volume, top and bottom face area, and height, respectively, and ρm represents the density of the matrix (titanium) phase. Significant differences can be observed, suggesting a severe deviation of as-built samples from the original CAD models. This can be attributed to various internal impurities, such as remnants of powder or clusters of slag (Figure 4a). Variability in the strut thickness and its deviation from the theoretical models (Figure 4b) is another source contributing to this inconsistency.

**Figure 3:**
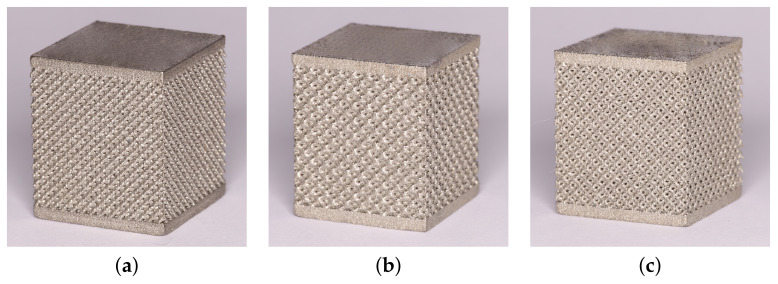
Examples of trabecular specimens made for compression tests: (**a**) D30-2, (**b**) DT-2, (**c**) RD30-2 (Table 1).

The trabecular part of each specimen, whether for compression or tension tests, was assumed to be a cube of 14 × 14 × 14 mm. This resulted in a variable number of basic units along the specimen edge (NofCellsEdge in Table 1). The resulting specimens intended for the compression tests appear in Figure 3. As seen, 1 mm thick bottom and top homogeneous bases were printed along with the trabecular part to allow for a uniform transmission of the applied load onto the porous structure.

**Figure 4:**
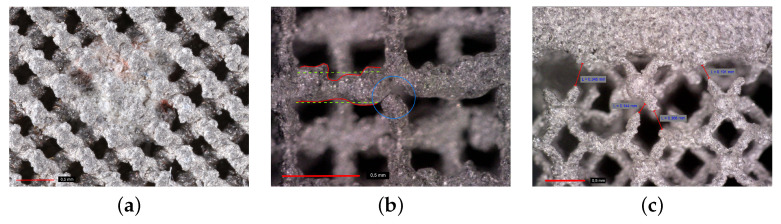
Defects of trabecular specimens: (**a**) clusters of slag inside trabecular structure, (**b**) discontinuities within trabecular structure and variability in strut thickness, (**c**) discontinuities (debonding) at interface between trabecular and homogeneous part (photographs provided by high-resolution camera Canon 6D Mark II).

It is worth mentioning that the specimens were printed gradually from the bottom to the top base. This becomes relevant when considering the major defects observed at the trabecular section–top base interface in Figure 4c. The most simple explanation is the rate of cooling of small trabeculae which considerably exceeds the one of a homogeneous part, causing detachment of struts from the base because of thermal shrinkage. This issue may seem less important in the production of the implant itself, providing the inner solid part is printed first, as no such discontinuities were observed at the bottom base–trabecular section interface. As well as interfacial debonding, other types of flaws may occur inside the porous structure, as demonstrated in Figure 4. It is our belief that these flaws are associated with the limitations of the used printing device and would not be observed for larger units with thicker struts. Therefore, application of trabecular structures is expected in the area of large joint replacement.

**Figure 5:**
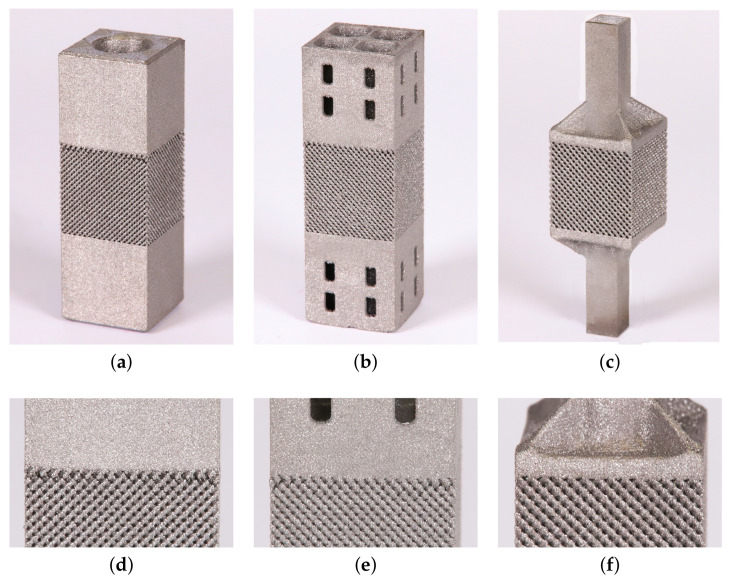
Trabecular specimens made for tension tests: (**a**) variant 1 (TT-V1), (**b**) variant 2 (TT-V2), (**c**) variant 3 (TT-V3), (**d**) visible interface defects TT-V1, (**e**) visible interface defects TT-V2, (**f**) no visible interface defects TT-V3.

While not as vital for compression tests, the specimen homogeneity plays a crucial role when loading the specimen in tension. A considerable effort has been invested into the preparation of specimens for tensile loading with emphasis on the elimination of interface flaws. The main concern when designing the specimen was to allow for quick heat escape when printing the clamping part while ensuring a sufficient strength and stiffness for this part to sustain the clamping pressure and allow for loading the trabecular part in pure tension. The process of development of individual variants is shown in Figure 5 identifying both unsuccessful (Figure 5a,b,d,e) and successful (Figure 5c,f) designs. Only the 3rd variant, TT-V3, with no visible interfacial defects was eventually tested.

**Figure 6:**
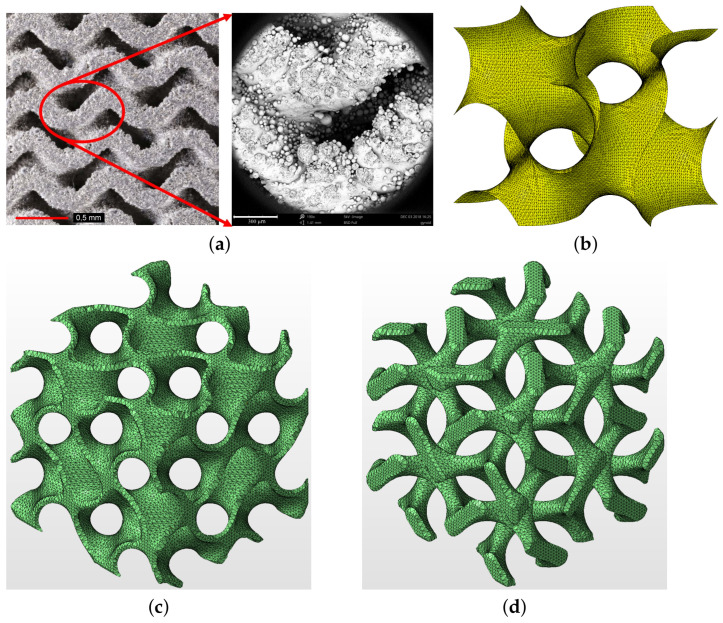
(**a**) Printed gyroid structure with no visible internal defects (left—photograph provided by high-resolution camera Canon 6D Mark II, right—microscopic image provided by electron microscope SEM Phenom XL), (**b**) example of one-cell gyroid surface with *t* = 0, (**c**) matrix phase gyroid with finite wall thickness $\delta^{w}=400$ μm, (**d**) inverted matrix phase gyroid assuming $\delta^{w}=400$ μm ((**b**–**d**) were generated using Autodesk Netfabb software).

Emerging limitations of 3D printing when applied to trabecular structures outweighed our interest towards gyroid microstructures. The arrival of TPMS structures considerably broadened the area of applications of cellular microstructures. Moving from sharp edges and corners typical of trabecular structures to smooth shells, forming the matrix of a cellular structure with opened mutually interconnected system of pores, which positively influences osseointegration and increases the inner structural stability, makes gyroids particularly attractive. Through mathematical formulation of a level set function (single gyroid) in terms of spatial coordinates x,y,z and edge length *l* of a cubic cell

(2) $$\sin\left(\frac{2\pi x}{l}\right)\cos\left(\frac{2\pi y}{l}\right)+\sin\left(\frac{2\pi y}{l}\right)\cos\left(\frac{2\pi z}{l}\right)+\sin\left(\frac{2\pi z}{l}\right)\cos\left(\frac{2\pi x}{l}\right)=t,$$

which permits a significant variation in microstructure morphology by suitably adjusting the isovalue *t* [9,42], the gyroid structures are poised for a major impact on future implant designs [1] regarding patient-specific needs. Furthermore, reasonable accuracy has been demonstrated between the original CAD designs and SLM-built Ti6Al4V structures [6], proving the good manufacturability of these structures. This is supported by our own observations suggesting that these structures are less prone to internal defects, as indicated in Figure 6a for illustration. To explain the presence of residuum particles, we mention the used sintering temperature of 1880 °C and a chamber temperature of around 20 °C. This represents a significant difference between the sintered and surrounding powder, causing the unsintered particles to weakly bond to a sintered surface. This effect may be reduced by decreasing the sintering temperature but at the expense of the quality of the printed specimen. Chemically removing these particles is another option but unnecessarily expensive, particularly for the performed mechanical tests.

An illustrative example of a one-cell single gyroid isosurface with t=0 is presented in Figure 6b while a two-cell matrix phase gyroid [9] with a finite, a priori defined wall thickness $\left(\delta^{w}\right)$ appears in Figure 6c. Note that an inverted matrix phase gyroid in Figure 6d more or less resembles the trabecular structure. Note also that the parameter *t* can be used directly to generate a wall system of a gyroid structure with a finite thickness of the wall, providing a double gyroid system represented by two single gyroids with an opposite orientation of the curvature of their surfaces [43]. However, this approach was not pursued in this study.

**Figure 7:**
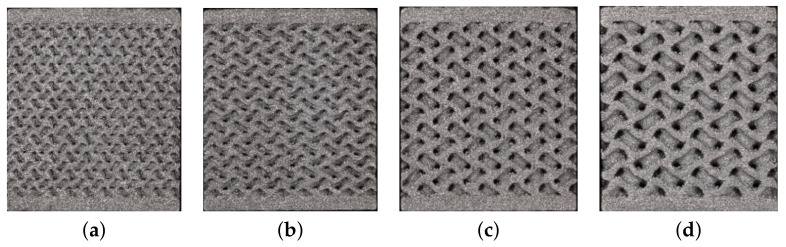
Basic cells of gyroid structures—Phase I: (**a**) G1-I, (**b**) G2-I, (**c**) G3-I, (**d**) G4-I.

We mentioned in the introductory part that an optimal pore size for efficient bone ingrowth ensuring a sufficient bone–implant bond is found in the range of 350–800 μm [20]. With this in mind, we first investigated the influence of pore size (Phase I) by considering four types of basic cells of variable size, and similarly for trabecular structures, adjusting both the pore size and the wall thickness while keeping comparable porosity. The wall thickness was about half the pore size. The corresponding data are available in Table 2. The specimen dimensions were assumed to be the same, both for compression and tension, as for the trabecular specimens, i.e., a 14 × 14 × 14 mm cube of a porous microstructure with 1 mm thick homogeneous bases. A graphical representation can be seen in Figure 7.

Next (Phase II), the effect of wall thickness, the parameter we were not able to address with trabecular structures, was examined. The geometrical details are summarized in Table 3. What is interesting to see, contrary to trabecular specimens, is that there is only a slight difference, with the exception of GII-2, between the theoretical porosity $n^{V}$ and the porosity $n^{m}$ of as-built specimens.

#### 2.1.2. Measurement and Evaluation of Selected Mechanical Properties

The compressive and tensile stress measurements were carried out in the displacement control regime with a loading rate of 1 mm/min using electromechanic MTS Alliance RT30kN and RT50kN loading machines for trabecular and gyroid microstructures, respectively. Given the specimen topology, the loading direction was assumed to be normal to the layers fabricated via 3D printing. The resulting measurements were visualized in terms of stress–strain diagrams. More specifically, the engineering strain was defined as the prescribed displacement divided by the specimen height, and the engineering stress was calculated by dividing the reaction force by the base area.

**Figure 8:**
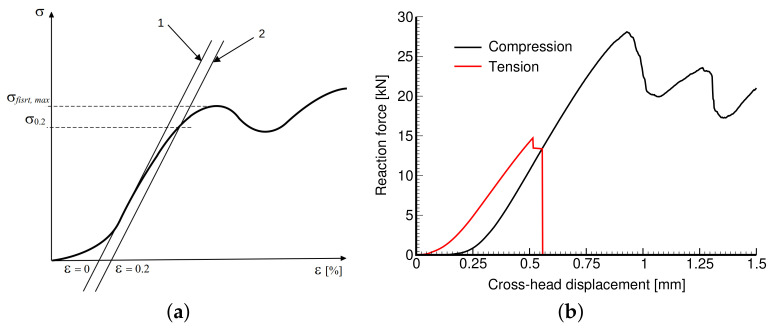
Engineering stress vs. engineering strain curve: (**a**) definition of basic mechanical parameters, (**b**) illustration of compressive and tensile stress–strain diagrams obtained experimentally.

The Young’s modulus *E*, the 2% yield strength in compression *σ*_0.2_, and the strength $\sigma_{first,max}$ representing the first maximum stress reached were derived in accordance with Figure 8a following the ISO 13314:2011 standard [44]. All specimens were loaded until failure. Figure 8b plots typical loading curves obtained from compression and tensile tests to highlight their fundamental differences. The Young’s moduli in compression and tension were derived from a linear part of the stress–strain diagrams as the slope of the tangent constructed just after passing the first inflex point on the curve. This also set the point of zero strain *ε*_0_ needed to identify a strength σ_0.2_ as the stress at which the stress–strain curve deviates by a strain of *ε* = 0.2% from the linear range. Note that both *σ*_0.2_ and $\sigma_{first,max}$ were estimated from the compression tests only.

### 2.2. Theoretical Formulation of Homogenization

A physical experiment is generally required whenever a new material or a product is expected to enter the world of engineering practice. However, such experiments are often expensive which hampers extensive parametric studies. On the contrary, such studies are inevitable, at least at the initial stage, when striving for new and optimal designs. A dental implant is a strong example [1]. In this regard, computational mechanics has proven beneficial.

Concerning the invention of porous microstructures intended for bio-applications, the simulations are typically directed towards a numerical reproduction of a physical experiment while accounting for all geometrical details of the tested specimen ([9,10,11,27] to cite a few). The resulting finite element model then typically calls for fine meshes with a large number of degrees of freedom which makes the analysis time consuming. Keeping in mind the periodicity of basic units, an attractive alternative is homogenization [30,32,33,34,45,46], often combined with multiscale analysis [47], especially when loading the material beyond its elastic limit [31,35,37]. We wish to adopt this approach and confirm its applicability in the context of the studied trabecular and gyroid structures. A brief theoretical background is provided while limiting attention to linear elasticity.

Given the loading conditions in the physical experiment outlined in Section 2.1.2, we proceed along the lines of strain-based periodic homogenization. Standard vector matrix notation is used herein with the boldface lowercase italic letter *a* representing an N×1 vector and the boldface capital letter A representing an N×M matrix. To that end, consider a representative volume element (RVE) in terms of a periodic unit cell *Y* (PUC). The periodic unit cell is assumed to be loaded on its external boundary ∂Y by the displacement field $u_{0}\left(x\right)$ which generates a macroscopically uniform strain *E* in an equivalent homogeneous medium which has the same overall (effective) properties as the original porous material. In view of the periodicity of PUC, the displacement and strain fields in PUC allow the following decomposition [30,34]

(3) $$u\left(x\right)=E\cdot x+u^{*}\left(x\right),\;\varepsilon \left(x\right)=E+\varepsilon^{*}\left(x\right)\;\mathrm{in}\;Y.$$

The fluctuation part $u^{*}$ of the displacement field *u* enters Equation (3) because of porosity. Note that $u^{*}$ is *Y*-periodic to ensure that

(4) $$\langle \varepsilon (x)\rangle =E,\;\langle \varepsilon^{*}\left(x\right)\rangle =\frac{1}{Y}\int_{Y}\varepsilon^{*}\left(x\right)\;dx=0,$$

where $\langle \cdot \rangle$ stands for volume averaging. Next, consider the Hill lemma in the form of principle virtual work and use the virtual displacement $\delta u\left(x\right)=\delta u^{*}\left(x\right)$ (***E*** is prescribed so that δE=0) to obtain

(5) $$\langle (\delta \varepsilon^{*}{\left(x)\right)}^{T}\sigma \left(x\right)\rangle =\langle \delta \varepsilon^{*}{\left(x)\right)}^{T}\rangle \langle \sigma (x)\rangle =0.$$

since $\langle \delta \varepsilon^{*}\rangle =0$ by Equation (4). To solve Equation (5), we adopt the standard displacement-based finite element method (FEM) and discretize the periodic cell *Y* into $N_{e}$ disjoint elements $Y_{e}$ respecting the material interfaces. The searched fluctuation part of the displacement field $u^{*}$ and the local strain field ε then assume the form

(6) $$u^{*}=N\left(x\right)r,\left(a\right)\;\varepsilon \left(x\right)=E+B\left(x\right)r,\left(b\right)$$

where N represents the shape functions for a given partition of the unit cell, B is the corresponding strain–displacement (geometric) matrix, and ***r*** is the vector of unknown degrees of freedom. Substituting Equation (6b) into Equation (5) yields for any kinematically admissible strains $\delta \varepsilon^{*}=B\delta r$ the associated system of algebraic equations

(7) Kr=f,

in terms of the stiffness matrix K of the system and the vector of generalized nodal forces *f* written as

(8) $$K=\overset{N_{e}}{\underset{e=1}{A}}K_{e},\;\;\mathrm{where}\;\;K_{e}=\frac{1}{Y}\int_{Ye}B_{e}^{T}(x)L_{e}B_{e}dx,$$

(9) $$f=\overset{N_{e}}{\underset{e=1}{A}}f_{e},\;\;\mathrm{where}\;\;f_{e}=\frac{1}{Y}\int_{Ye}B_{e}^{T}(x)L_{e}Edx,$$

where $L_{e}$ is the material stiffness matrix of the *e*-th element $\left(\sigma_{e}\left(x\right)=L_{e}\varepsilon_{e}\left(x\right)\right)$, and the operator error represents a standard assembly (localization) of contributions from individual elements.

System (7) can be used to provide the finite element approximation of the coefficients of the effective stiffness matrix $L^{\mathrm{hom}}$ as volume averages of the local fields derived from the solution of successive elasticity problems [34]. Considering a three-dimensional body, we load PUC, in turn, by each component of *E* while the other components vanish. The volume stress averages $\left(\langle \sigma (x)\rangle =\Sigma \right)$ normalized with respect to *E* then fill individual columns of $L^{\mathrm{hom}}$ to obtain the macroscopic constitutive law as

(10) $$\Sigma =L^{\mathrm{hom}}E.$$

## 3. Results

The porous microstructures introduced in Section 2.1.1 are compared both quantitatively and qualitatively on the basis of selected mechanical properties. The results from the proposed experimental program are discussed first in Section 3.1. The measured elastic response further serves to validate the effective elastic properties predicted numerically in Section 3.2 on the grounds of computational homogenization (Section 2.2).

### 3.1. Experimentally Derived Mechanical Properties

**Figure 9:**
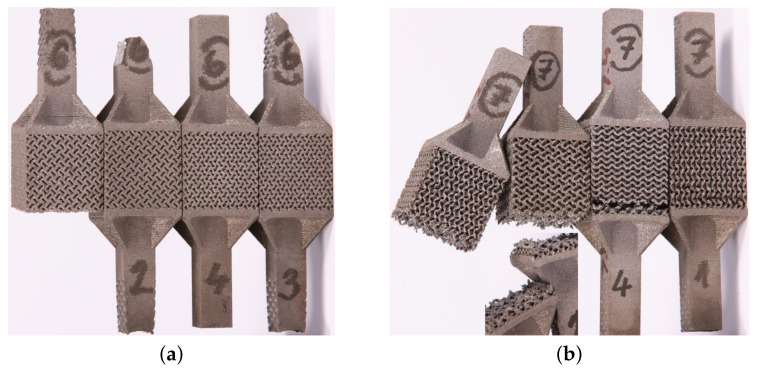
Fractured specimens undergoing tension: (**a**) failure at gripping section (samples 2, 3—porous structure corresponds to GII-2), (**b**) location of fracture surfaces near top base (porous structure corresponds to GII-3, specimens are positioned upside-down from the printing direction point of view).

In Section 2.1.1, we proposed a route to fabricate specimens we expected to provide the response of printed microstructures in tension. However, the measurements of both trabecular and gyroid specimens were highly inconsistent. A number of specimens experienced failure within the gripping section or in the vicinity of the top base. In general, the fracture was brittle and the fracture surface revealed a number of internal discontinuities, particularly for trabecular specimens, not observed by initial inspection (Figure 5f). Figure 9 shows some of the fractured specimens for illustration.

Testing the specimens prepared entirely by the SLM printing technique in tension thus remains a challenge and at the present time we are not able to offer reliable results. Therefore, the remainder of this section is devoted to compression, concentrating on the effective Young’s modulus, yield strength *σ*_0.2_, and the first maximum stress $\sigma_{first,max}$, all measured along the printing direction. For each microstructure in Table 1, Table 2 and Table 3, three specimens were fabricated and tested following the procedure described in Section 2.1.2.

#### 3.1.1. Trabecular Specimens in Compression

**Figure 10:**
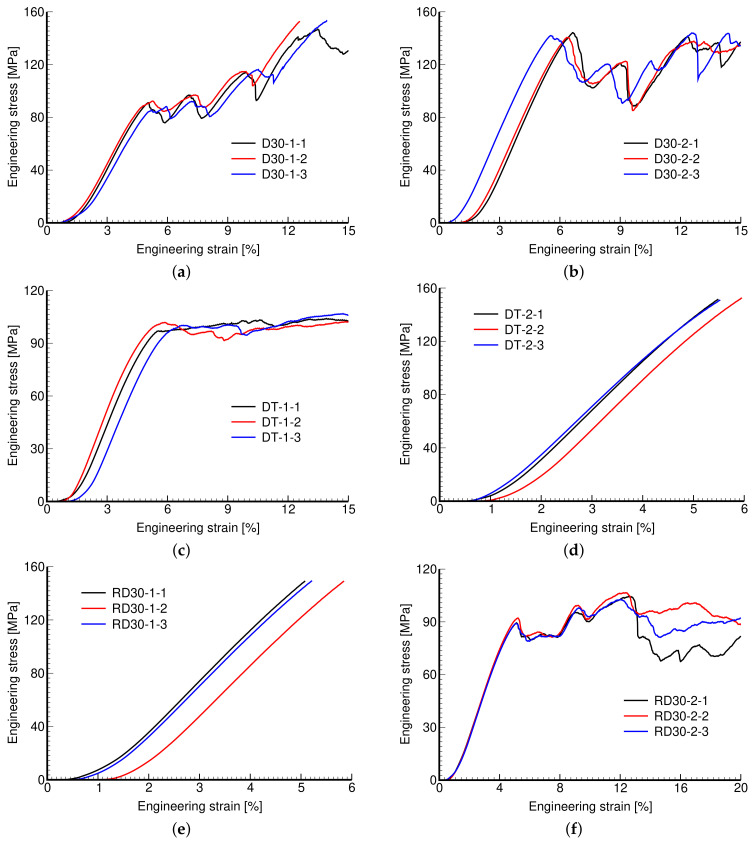
Engineering stress vs. engineering strain curves: (**a**) D30-1, (**b**) D30-2, (**c**) DT-1, (**d**) DT-2, (**e**) RD30-1, (**f**) RD30-2.

Figure 10 plots the measured stress–strain diagrams. While the consistency of results pertinent to a given microstructure is evident, we observe a considerable variability across individual microstructures. Exceeding the maximum allowable reaction force of 30 kN in the MTS RT30kN loading system terminated the experiment for some microstructures even prior to reaching $\sigma_{first,max}$ (see Figure 10d,e).

**Figure 11:**
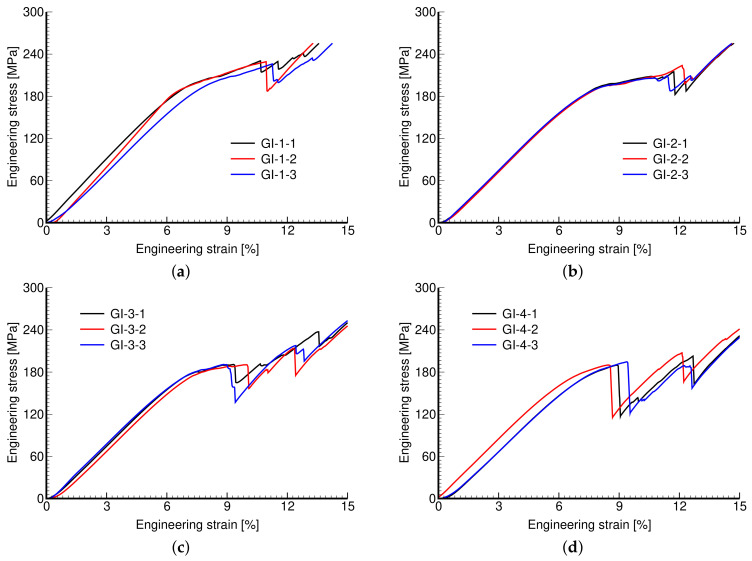
Engineering stress vs. engineering strain curves: (**a**) GI-1, (**b**) GI-2, (**c**) GI-3, (**d**) GI-4.

**Figure 12:**
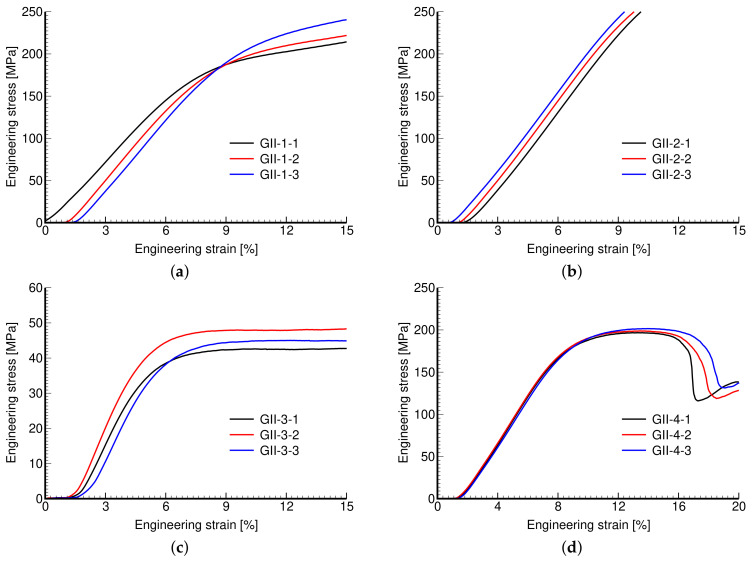
Engineering stress vs. engineering strain curves: (**a**) GII-1, (**b**) GII-2, (**c**) GII-3, (**d**) GII-4.

It is evident that the response is highly affected by the microstructural details, such as the pore size and thickness of struts, including the geometry of the basic unit and not just porosity. This is confirmed by the measured mechanical properties shown in Table 4. The experimental results presented in Table 4 and Table 5 are averages from three measurements (Figure 10, Figure 11 and Figure 12). This also applies to actual porosity $n^{m}$ slightly differing across specimens, compare, for example, the measured Young’s modulus corresponding to D30-1 and DT-1 or to D30-2 and DT-2 microstructures, with both pairs having comparable porosity, but differing in geometrical details, pore size, and strut thickness. However, there was relative difficulty in removing the powder agglomerate residue, resulting in printed samples deviating quite strongly from the theoretical models, identified by the differences in $n^{m}$ and $n^{V}$, which puts the reliability of the measured results into question.

The values of Young’s modulus support the examined microstructures, which are all found within the range of 2.7–9.1 GPa, typical of spongious bones. If striving for a low stiffness while maintaining a sufficient strength then the Rhombig RD30-1 system would be the most effective. What could be the obstacle for applications in the area of small dental implants is the relatively small thickness of struts below 300 μm, being not only at the edge of production limitations but also causing a large number of capillary defects. These are unacceptable because they represent a potential source of broken microparticles, causing necrosis and consequently an aseptic release of implants. Thus, for small implants, the examined trabecular structures look like a dead end. A different route is therefore needed, e.g., the use of gyroid systems, as discussed next.

#### 3.1.2. Gyroid Specimens in Compression

The measured stress–strain curves of both phases of investigation are shown in Figure 11 and Figure 12. Again, the reproducibility of individual microstructures is confirmed.

Remember that in Phase I, we aimed to create microstructures of a variable pore size while keeping the same porosity, simply by rescaling the dimensions of the basic unit, i.e., increasing the pore size generated larger units with thicker walls. The responses of these microstructures are very similar. This is quantitatively supported by the values of tracked mechanical properties in Table 5.

Phase II, on the other hand, addressed the impact of variable wall thickness while keeping the pore size and dimensions of the basic unit the same. Thicker walls thus reduce the overall porosity with obvious consequences manifested by increased stiffness (mild) and strength (significant). This is particularly evident for large pores in Figure 12c,d and from the corresponding values of mechanical properties in Table 5. As seen in Figure 12b, the strength properties of the GII-2 microstructure were not determined because of early termination of the test when the maximum reaction force exceeded the machine limit of 50 kN (MTS RT50kN).

#### 3.1.3. Comparing Trabecular and Gyroid Structures

**Figure 13:**
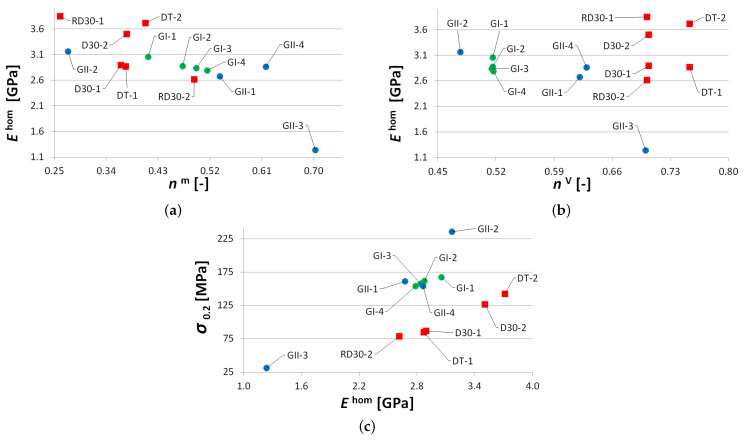
Comparing mechanical properties of trabecular and gyroid structures: (**a**) Young’s modulus vs. actual porosity $n^{m}$, (**b**) Young’s modulus vs. theoretical porosity $n^{V}$, (**c**) yield strength in compression vs. Young’s modulus.

Some specific differences in the response of trabecular and gyroid structures have already been put forward here. These are graphically identified in Figure 13. As mentioned, the properties of the studied trabecular structures strongly depend on the geometry of the basic unit and, unlike gyroid structures, one can hardly draw a simple correlation with the porosity only (Figure 13a,b). It can be seen in Figure 13c that a gyroid structure attains a considerably higher strength compared to a trabecular structure with identical Young’s modulus. This is advantageous, especially regarding final implant stability. Note that, with the exception of the GII-3 specimen possessing the highest porosity, the measured Young’s moduli of gyroid specimens show a relatively mild deviation over a substantial range of actual porosities, $n^{m}$ = 0.41–0.63.

On the contrary, comparable Young’s moduli of trabecular and gyroid structures may call these results into question as a much stiffer response would be, in general, expected for gyroid structures. This issue is examined computationally in Section 3.2.

On top of the superior mechanical response, the gyroid structures possess additional assets, at least in the context of small dental implants, including smooth transition of the solid phase, free of sharp corners and edges, which promotes osseointegration. Compared to trabecular structures, the gyroid structure is less prone to internal defects and enables higher variability in the pore size–wall thickness ratio which is the principal issue in the construction of strong, stable, and safe implants. The trend in the future development of trabecular structures is thus seen more in the application of inverse gyroid microstructures, such as the one in Figure 6d possessing all previously mentioned benefits of gyroid structures.

### 3.2. Effective Elastic Properties Predicted by Homogenization

The experimentally observed behavior of trabecular and gyroid structures in the linear range is compared here to the results of numerical simulations. We wish to promote homogenization to save computational time and open the way to an efficient search for optimal designs which meet the specific conditions of a patient.

**Figure 14:**
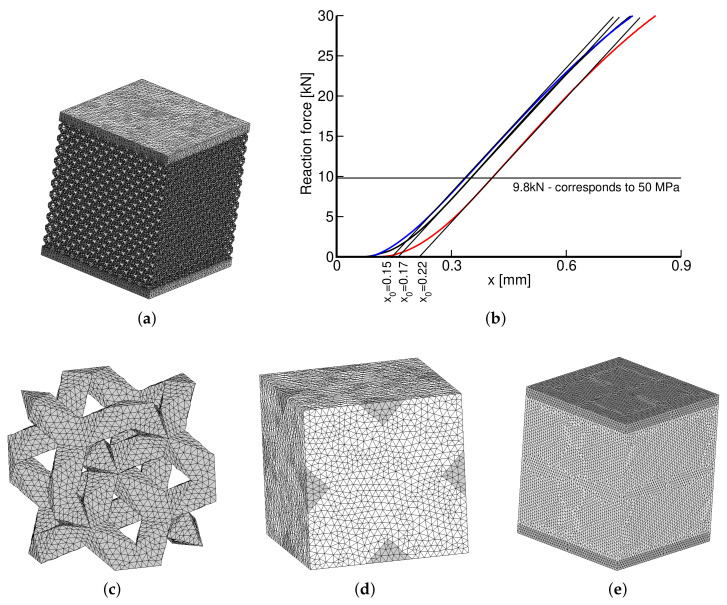
(**a**) Detailed FE model of 1/8 of DT-2 specimen, (**b**) measured loading curves (*x*—cross-head displacement), (**c**) solid part of DT-2 PUC (recall Figure 2b), (**d**) PUC finite element mesh, (**e**) coarse FE model of homogenized specimen.

Before examining individual morphologies, we present one comparative study on a Dode Thick type of microstructure. Figure 14a shows a detailed finite element mesh (DM) of a computational model to reproduce the actual experiment computationally. Because of symmetry, only one eighth of the test sample with standard symmetry constraints was eventually considered in simulations. The coarse homogeneous finite element model (HM) in Figure 14e with assigned effective properties derived from homogenization was expected to provide an identical response. The effective properties were derived from homogenization employing Equations (7)–(10) and the finite element model of a periodic unit cell in Figure 14c,d. Constant strain four-node tetrahedral elements were used in all simulations. The mesh in Figure 14d is periodic, allowing us to enforce the periodic boundary conditions, i.e., the same fluctuation displacements on opposite faces, by simply assigning the same code numbers to corresponding nodes (degrees of freedom). The corner nodes are fixed. The mesh details, including the computational time, appear in Table 6. All computations were performed on a powerful computer equipped with four processors, Intel(R) Xeon(R) CPU E5-2630 v3 2.40 GHz with RAM 128 GB.

The results of individual simulations, corresponding to the Young’s modulus E=118 GPa and the Poisson ratio *ν* = 0.3 of the solid phase obtained from nanoindentation, are shown in Table 7. The two macromodels in Figure 14a,e were loaded by a uniform pressure of 50 MPa, well below the elastic limit of all trabecular structures, recall the values of *σ*_0.2_ in Table 4. This is also illustrated in Figure 14b plotting the measured loading curves. The parameter $x_{0}$ identifies the starting point to measure the sample shortening $\Delta h=x-x_{0}$ where *d* is the applied cross-head displacement. Note that Δ*h* in the last row in Table 7 is again averaged over the three measurements.

To arrive at a better agreement of Δ*h* between DM and the experiment would require even finer mesh or higher order elements. This would also reduce the model stiffness but at the expense of an already long computational time. The computational model was built to acquire the theoretical porosity of $n^{V}=0.75$. Comparing the predicted and measured Young’s moduli suggests that internal impurities within the porous phase are bonded to the matrix phase only weakly, providing no additional stiffening. From an osseointegration point of view this is, however, unacceptable, further urging future improvement of the current printing capabilities. Nevertheless, the results still indicate reliability of the proposed homogenization technique which is further validated in the next two sections.

Prior to that, it is worth mentioning that homogenization, as opposed to running a single virtual test, allows us to calculate the entire stiffness matrix of the equivalent homogeneous medium and consequently to disclose the potential anisotropy of a given microstructure. To support a single value of the Young’s modulus in Table 7, we note in advance that both the trabecular and gyroid geometries are macroscopically isotropic.

#### 3.2.1. Trabecular Periodic Unit Cell

**Figure 15:**
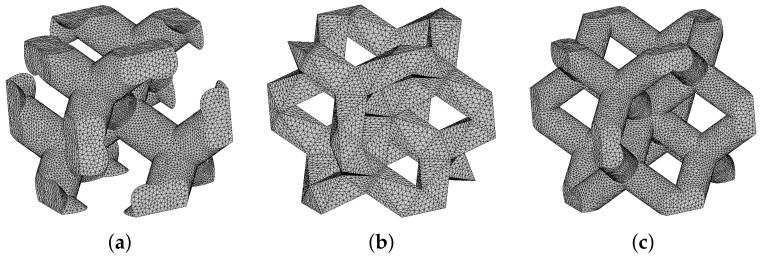
Finite element meshes of basic cells of trabecular structure: (**a**) D30-1, (**b**) DT-1, (**c**) RD30-1.

Table 8 summarizes the results of numerical homogenization, adopting the periodic unit cells in Figure 2. Sufficiently fine meshes were used to arrive at porosities ${\tilde{n}}^{V}$ comparable to their theoretical values $n^{V}$ in Table 4. One particular example for each system is plotted in Figure 15 for illustration. Because of macroscopic isotropy, we again present only the Young’s modulus and Poisson ratio. From a theoretical point of view, a single unit cell for each microstructure would be sufficient, as demonstrated by the homogenized values of material properties that are almost identical for a given system. The minor differences are just attributed to an error caused by discretization. This is particularly evident for the DT-1 and DT-2 systems, as a coarser mesh was deliberately used for the DT-2 system to show the relatively weak dependence on the mesh refinement, at least in the elastic range. In light of this, the results for different PUCs are reported merely to allow for a direct comparison with experimental values listed in the 2nd column in Table 8.

While the opening paragraphs in Section 3.2 served to validate numerical predictions via experiments, the results in Table 8 require addressing this issue from the opposite direction. The numerical predictions fully uncovered previously mentioned shortcomings of 3D printing when creating trabecular structures of such small sizes. This is particularly seen for diamond and rhombic structures with smooth cylindrical struts, which were difficult to manufacture for the assumed thicknesses of struts $\delta^{S}$ (Table 1 and Figure 4).

#### 3.2.2. Gyroid Periodic Unit Cell

After having difficulty in generating periodic meshes for a gyroid type of PUC, we abandoned the standard FEM formulation and performed the homogenization analysis in the framework of the extended finite element method (X-FEM) [48]. Because X-FEM enables an application of regular meshes, e.g., standard brick elements, which do not have to conform to material boundaries, the analysis of such complex geometries becomes less problematic [34,49].

**Figure 16:**
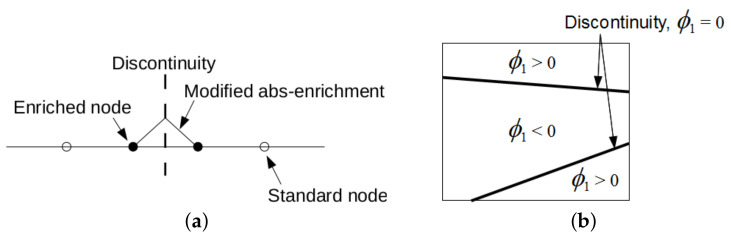
(**a**) Modified abs-enrichment for 1D problem, (**b**) element crossed by two interfaces of the same material phase.

In X-FEM, standard approximation of the displacement field in Equation (6a) is augmented by introducing the enrichment function ψ(x)

(11) $$u^{*}\left(x\right)=\sum_{i\in I}N_{i}\left(X\right)r_{i}+\sum_{j\in I^{*}}N_{j}\left(x\right)\psi \left(x\right)a_{j},$$

where $N_{i}$ are the standard shape functions, *I* represents the total number of finite element nodes in the analyzed domain, $I^{*}\subset I$ gives the number of nodes for which the support is split by the interface, and $a_{j}$ are the additional degrees of freedom. The present implementation follows [48] and assumes ψ(x) in the form

(12) $$\psi \left(x\right)=\sum_{i\in J}\left|\phi_{i}^{\mathrm{LS}}\right|N_{i}\left(x\right)-\left|\sum_{i\in J}\phi_{i}^{\mathrm{LS}}N_{i}\left(x\right)\right|,$$

where $\phi_{i}^{\mathrm{LS}}$ denotes the level set value in the node *i*. A one-dimensional format of this function is plotted in Figure 16a. The nodal values $\phi_{i}^{\mathrm{LS}}$ represent the signed distance of the element node to the interface with either a positive or a negative value depending on the material to which it belongs, as shown in Figure 16b. This function then locates interfaces implicitly as a union of points for which it attains a zero value (zero-level).

As it goes beyond the scope of the present study, we do not develop this subject any further and refer the interested reader to [34] where all details, including the formulation of a proper integration rule and implementation of periodic boundary conditions for both standard and additional degrees of freedom, are available.

#### Validation and Verification of X-FEM Implementation

**Figure 17:**
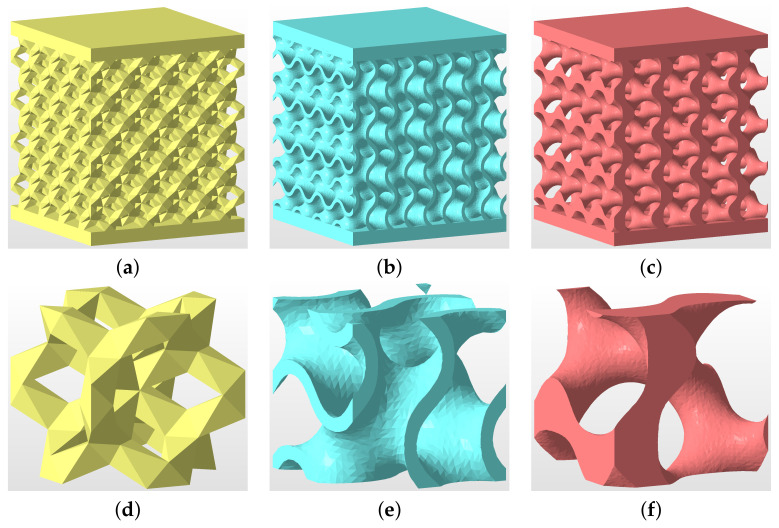
(**a**–**c**) Geometry of specimens tested experimentally in uniaxial compression: (**a**) DT-2, (**b**) GS, (**c**) GT; (**d**–**f**) basic unit cells: (**d**) DT-2, (**e**) GS, (**f**) GT.

To support the numerically predicted Young’s moduli of titanium specimens from Phase I (GI) and II (GII) examined experimentally in Section 3.1.2, we first present a short study on specimens made from plastic material as their preparation is both time and cost effective. To this end, three particular microstructures in Figure 17 were printed using the Sinterit Lisa Pro printer. It adopts a selective laser sintering (SLS) method to fabricate samples from a polyamid powder with a tensile strength of about 41 MPa.

The theoretical porosity of all specimens is $n^{V}=0.75$. The first microstructure in Figure 17a corresponds to DT-2 (Dode Thick), while the gyroid structures in Figure 17b,c were derived from Equation (2) setting t=0 (sheet gyroid, GS) and t=0.78 (trabecular gyroid, GT), respectively. The wall thickness of the sheet gyroid in Figure 17b was assumed to be $\delta^{w}=0.5$ mm. All specimens consisted of a 4 × 4 × 4 array of basic units cells, as shown in Figure 17d–f, each having the dimensions of 6.28 × 6.28 × 6.28 mm. Such samples proved sufficient to represent a periodic microstructure.

**Figure 18:**
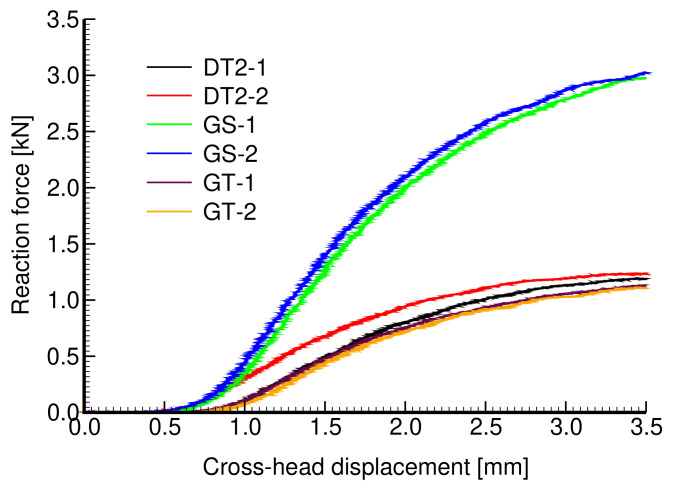
Experimentally derived force×displacement curves for specimens in Figure 17a–c.

The results of a uniaxial compression test are plotted in Figure 18. Four specimens were tested to check reproducibility (only two are shown for the clarity of graphical presentation). The extracted Young’s moduli obtained from the approach described in Section 2.1.2 and assuming the elastic parameters of the solid phase E=850 MPa and ν=0.33 are shown in Table 9. These results confirm a considerably higher strength of a sheet gyroid in comparison to a trabecular structure, as already observed for titanium specimens in Section 3.1.2 and Section 3.1.3. Moreover, the sheet gyroid also shows, as one would expect, a significantly higher stiffness, a feature not experimentally found for titanium specimens.

**Figure 19:**
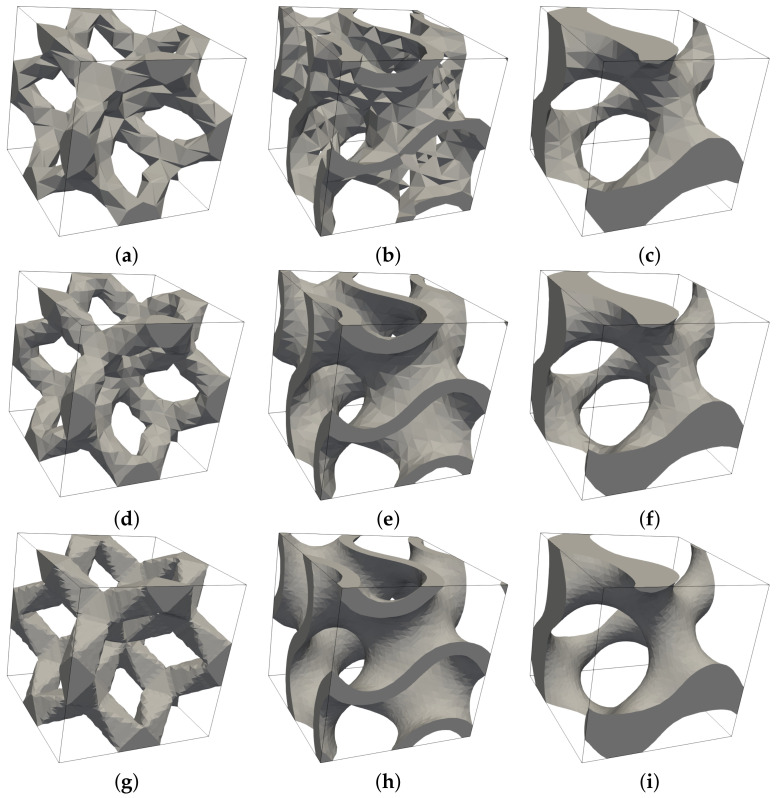
Influence of mesh refinement on approximation of solid phase by X-FEM: (**a**–**c**) subdivision into 10 × 10 ×10 brick elements, (**d**–**f**) subdivision into 15 × 15 × 15 brick elements, (**g**–**i**) subdivision into 35 × 35 × 35 brick elements.

The resulting effective elastic moduli derived both experimentally and from numerical homogenization are shown in Table 9. To arrive at X-FEM predictions, a relatively fine subdivision of periodic cells into 35 × 35 × 35 eight-node brick elements was used. The need for a sufficient refinement to correctly capture the shape and volume of the solid phase is demonstrated in Figure 19.

A relatively good match between measured and predicted effective moduli, given a non-negligible scatter of experimental data and uncertainty in the value of the Young’s modulus of sintered powder, validates the X-FEM implementation. This is further verified by standard FEM analysis carried out for the DT-2 structure. A better match can be expected if increasing the numbers of cells in tested specimens, thus reducing the edge effect not present in homogenization.

#### Titanium Specimens GI and GII

Table 10 compares the results found from experimental measurements (Section 3.1.2) and X-FEM homogenization considering again the subdivision of PUC into 35 × 35 × 35 brick elements. It is seen that the volume fraction of pores approximated by X-FEM ${\tilde{n}}^{V}$ and the theoretical value $n^{V}$ are mostly identical, supporting the assumed degree of refinement. The corresponding total number of degrees of freedom appears in the last column of this table. While the number of elements is much smaller than in standard FEM analyses (Table 8) the computational burden is comparable. However, the simplicity in preparing the finite element mesh with no need to comply with material boundaries is clear.

As expected from the previous study performed on plastic specimens, the homogenization suggests a considerably stiffer response than observed experimentally for this type of specimen. Whether this can be attributed to the quality of the printed samples is unclear. We hope to reconcile this discrepancy with the help of micro-CT scanning, enabling us to uncover potential defects inside the microstructure not visible to the naked eye. This is an ongoing research effort and the results will be presented elsewhere.

## 4. Discussion

Research activities in the field of biomedical titanium implants are quite intense. Developments of novel designs have been stimulated by a rapid increase in 3D printing capabilities to manufacture implants of desired strength and stiffness [50]. However, practical applications in some areas are still rather scarce due to the limitations of the current printing technology and demanding legislation processes for new products on the market [51]. Small dental implants fall into this category. Therein, the size of a typical microstructural load-bearing element is the principal obstacle in producing reliable and patient-safe implants [52]. Some of the issues regarding the needs for both experimental and theoretical studies are presented in this paper.

Our attention was devoted to porous microstructures manufactured by the SLM technique. Limitations of the adopted 3D printing technology were found when examining a particular class of trabecular structures, which showed a number of internal defects. This resulted mainly from the need for printing struts of a very small size, at the limit of the printer’s capabilities, partially influenced by the grain size of the used Ti6Al4V powder. To eliminate some of the defects, a novel design of specimens, particularly those loaded in tension, was proposed. However, the results of an extensive experimental program, which also included an inspection of microstructural details, suggested the application of trabecular structures for implants of larger sizes than those expected in dental medicine.

Therefore, we turned our attention to a gyroid structure. At first glance, the produced specimens have shown much less susceptibility to internal defects with final porosity matching quite well that of a theoretical model. We also observed a better consistency in the quality of the produced specimens in comparison to trabecular ones, which was also supported by a smaller variability in the measured mechanical properties. As expected, the gyroid structures showed a significant increase in strength when compared to values achieved for trabecular samples with comparable porosities (Table 4 and Table 5). It is worth mentioning that trabecular samples produced as an inverted matrix gyroid (Figure 6d) were not examined in the present study. What appears less credible is the measured stiffness being within the range of trabecular specimens. At this point, we should strengthen the need for the close interaction of experiments and numerical simulations to either support each other or uncover potential errors of individual research activities. This is in accordance with what Drucker postulated in [53], “Theory awaits experiment and experiment awaits theory in a wide variety of fields. Often the two must go hand in hand if significant progress is to be made.”

In this study, we approached the computational effort in the framework of first-order computational homogenization [42,54]. This computational strategy has proven reliable and efficient in many areas of engineering, especially if the material microstructure is deemed periodic [34]. Standard finite element simulations on trabecular specimens were performed first to promote their applicability. Difficulty in constructing periodic meshes for gyroid structures shifted our attention to the extended finite element method (X-FEM) [55]. A short study on specimens made of plastic material not only confirmed the correct implementation of the method but suggested a much stiffer elastic response of the sheet gyroid compared to trabecular structures (see also [42] for similar findings). That finding conflicted with a relatively low stiffness of titanium specimens offered by experimental measurements but was aligned with numerical predictions, which suggested higher stiffness of an order of magnitude. At this point, however, one cannot simply reject one of the two results and so further research is needed. We believe that computational microtomography used with loaded specimens [56,57] will shed light on this subject as this approach has already been successfully used in [58] to inspect the quality of 3D printed microstructures. For application to polymer-based gyroid structures, see, for example, [54]. This research activity is currently underway, and the results will be presented separately. On the contrary, it is worth mentioning that a reasonable agreement between experimental and direct numerical simulations of full-size specimens has been achieved for a number of sheet gyroid microstructures made of both polymer and titanium material ([9,59] to cite a few).

With the above points in mind, future research activity is expected to concentrate on the design and modeling of a sheet gyroid in both pre- and post-failure regimes [59,60]. To reconcile the observed differences in experimental measurements and computational predictions, computational microtomography will be used. The expected limitation is seen in the sufficient accuracy to represent the most relevant flaws in the printed geometry and to allow for the generation of a reliable computational model [61]. The associated computational modeling based on X-FEM, a tool which proved powerful in the homogenization of complex microstructures [48,49,62], will then require an efficient C++ implementation to handle the memory limitations of the present Matlab implementation. Such a direct combination of experimental and computational research should help to avoid conceptual, systematic, or random errors.

## 5. Conclusions

An extensive experimental and computational program was performed to investigate the response of small porous microstructures representing the outer section of a dental implant. The specimens were manufactured with the range of pore size (300–800 μm) assumed optimal from the bone cell ingrowth point of view [12,23]. The associated thicknesses of struts and walls in the range of 150–300 μm of trabecular and sheet gyroid specimens, respectively, revealed limitations of the adopted SLM-based 3D printing technology. This was illustrated by flows at the porous structure–solid base interface, attributed to a rapid heat exchange during production (Section 2.1.1). This issue deserves particular attention as it currently represents a weak point in the development of novel dental implants [1]. However, progress has been made in the design of specimens for tensile loading, which seems to be unique according to the authors’ knowledge and opens the door to testing this material in a more general loading regime.

The impact of these flaws on the material response was examined by comparing the results of laboratory measurements with virtual (computational) experiments performed on ideal mircostructures. To this end, a powerful X-FEM-based homogenization was promoted to reduce computational cost and remove obstacles arising when meshing such complex geometries. This study showed a relatively minor effect of the observed flaws on the response of trabecular specimens. On the contrary, the considerable difference between measurements and simulations encountered for gyroid specimens supports the need for physical experiments, which are much too often substituted by considerably cheaper numerical analyses [63], which in turn may falsely supplant some physical facts, e.g., the potential flaws we did not originally expect for the printed gyroid structures.

## Figures and Tables

**Table 1:** Types and geometry of basic trabecular cell units (D30—diamond, relative density 30%, DT—Dode Thick, RD30—rhombic dodecahedron, relative density 30%).

Unit Type	*L* [μm]	*δ*^s^ [μm]	*d* [μm]	$n^{m}$ [-]	$n^{V}$ [-]	NofCellsEdge
D30-1	750	200	350	0.37	0.70	18
D30-2	1000	260	450	0.38	0.70	14
DT-1	1000	200	500	0.37	0.75	14
DT-2	1250	250	630	0.41	0.75	11.5
RD30-1	1250	230	640	0.26	0.70	11.5
RD30-2	1500	290	800	0.49	0.70	9.5

**Table 2:** Types and geometry of basic gyroid cell units—Phase I.

Unit Type	*L* [μm]	*d* [μm]	$n^{m}$ [-]	$n^{V}$ [-]	NofCellsEdge
GI-1	1400	400	0.41	0.52	10
GI-2	1800	450	0.47	0.52	7.78
GI-3	2400	700	0.50	0.52	5.83
GI-4	3000	800	0.52	0.52	4.67

**Table 3:** Types and geometry of basic gyroid cell units—Phase II.

Unit Type	*L* [μm]	*δ*^s^ [μm]	*d* [μm]	$n^{m}$ [-]	$n^{V}$ [-]	NofCellsEdge
GII-1	1800	150	450	0.54	0.62	7.78
GII-2	1800	250	450	0.27	0.48	7.78
GII-3	2700	150	750	0.70	0.70	5.18
GII-4	2700	250	750	0.62	0.63	5.18

**Table 4:** Mechanical properties of trabecular structures from experiments.

Unit Type	*E* [GPa]	*σ*_0.2_ [MPa]	*σ_first,max_* [MPa]	$n^{m}$ [-]	$n^{V}$ [-]
D30-1	2.88	86.7	88.7	0.37	0.70
D30-2	3.51	126.5	141.9	0.38	0.70
DT-1	2.84	84.7	98.2	0.37	0.75
DT-2	3.71	142.2	-	0.41	0.75
RD30-1	3.82	-	-	0.26	0.70
RD30-2	2.63	78.4	90.2	0.49	0.70

**Table 5:** Mechanical properties of gyroid structures from experiments.

Unit Type	*E* [GPa]	*σ*_0.2_ [MPa]	*σ_first,max_* [MPa]	$n^{m}$ [-]	$n^{V}$ [-]
GI-1	3.05	166.9	228.5	0.41	0.52
GI-2	2.87	161.6	214.5	0.47	0.52
GI-3	2.84	157.8	190.7	0.50	0.52
GI-4	2.77	154.1	191.4	0.52	0.52
GII-1	2.67	161.3	-	0.54	0.62
GII-2	3.16	235.8	-	0.27	0.48
GII-3	1.24	31.0	-	0.70	0.70
GII-4	2.86	154.1	199.0	0.62	0.63

**Table 6:** Details of finite element models.

Mesh Details	Detailed Model	Homogeneous Model	PUC
Number of nodes	304,289	3461	25,061
Number of elements	1,016,821	17,285	110,427
Computational time	2 h and 13 min	6 s	34 s

**Table 7:** Results from initial comparative study on DT-2 geometry.

Model	*E* [GPa]	*ν* [-]	Δ*h* [mm]
Detailed model	4.11	-	0.17
Homogeneous model	-	-	0.18
PUC	3.56	0.244	-
Experiment	3.71	-	0.19

**Table 8:** Effective elastic properties of trabecular structures from homogenization.

Unit Type	Experiment	Homogenization				
	*E* [GPa]	*E* [GPa]	*ν* [-]	${\tilde{n}}^{V}$ [-]	Num. Nodes	Num. Elems
D30-1	2.88	4.42	0.296	0.704	50,045	263,404
D30-2	3.51	4.41	0.295	0.705	49,551	260,533
DT-1	2.84	3.39	0.246	0.754	47,573	232,019
DT-2	3.71	3.56	0.244	0.756	25,061	110,427
RD30-1	3.82	5.94	0.296	0.703	61,172	329,417
RD30-2	2.63	5.97	0.277	0.703	60,916	328,003

**Table 9:** Effective elastic properties of structures in Figure 17 from measurements and homogenization.

Unit Type	Experiment	Homogenization	
	*E* [MPa]	*E* [MPa] (X-FEM)	*E* [MPa] (FEM)
DT-2	27.3 ± 3.8	26.4	25.9
GS	28.7 ± 1.8	33.2	-
GT	72.4 ± 6.1	82.9	-

**Table 10:** Effective elastic properties of gyroid structures from homogenization.

Unit Type	Experiment	Homogenization			
	*E* [GPa]	*E* [MPa]	*ν* [-]	${\tilde{n}}^{V}$ ($n^{V}$) [-]	Num. Dofs.
GI	2.88 (mean)	28.6	0.29	0.51 (0.52)	191,043
GII-1	2.67	19.6	0.29	0.62 (0.62)	194,436
GII-2	3.16	32.3	0.28	0.47 (0.48)	189,432
GII-3	1.24	14.0	0.31	0.70 (0.70)	195,636
GII-4	2.86	18.2	0.30	0.63 (0.63)	194,724

## Data Availability

Not applicable.
